# Economic analysis of the robotic approach to inguinal hernia versus laparoscopic: is it sustainable for the healthcare system?

**DOI:** 10.1007/s10029-024-03006-y

**Published:** 2024-03-20

**Authors:** F. Hinojosa-Ramirez, L. Tallon-Aguilar, J. Tinoco-Gonzalez, A. Sanchez-Arteaga, F. Aguilar-Del Castillo, I. Alarcon-Del Agua, S. Morales-Conde

**Affiliations:** 1 Department of Surgery, Hospital Universitario Virgen del Rocío, Av Manuel Siurot S/N, 41013 Seville, Spain; 2 Surgery Department, University of Seville, Avda. Doctor Fedriani, s/n, 41009 Seville, Spain

**Keywords:** Inguinal hernia, Robotic approach, Laparoscopic approach, Cost-analysis, Cost-effective

## Abstract

**Introduction:**

There has been a rapid proliferation of the robotic approach to inguinal hernia, mainly in the United States, as it has shown similar outcomes to the laparoscopic approach but with a significant increase in associated costs. Our objective is to conduct a cost analysis in our setting (Spanish National Health System).

**Materials and methods:**

A retrospective single-center comparative study on inguinal hernia repair using a robotic approach versus laparoscopic approach.

**Results:**

A total of 98 patients who underwent either robotic or laparoscopic TAPP inguinal hernia repair between October 2021 and July 2023 were analyzed. Out of these 98 patients, 20 (20.4%) were treated with the robotic approach, while 78 (79.6%) underwent the laparoscopic approach. When comparing both approaches, no significant differences were found in terms of complications, recurrences, or readmissions. However, the robotic group exhibited a longer surgical time (86 ± 33.07 min vs. 40 ± 14.46 min, *p* < 0.001), an extended hospital stays (1.6 ± 0.503 days vs. 1.13 ± 0.727 days, *p* < 0.007), as well as higher procedural costs (2318.63 ± 205.15 € vs. 356.81 ± 110.14 €, *p* < 0.001) and total hospitalization costs (3272.48 ± 408.49 € vs. 1048.61 ± 460.06 €, *p* < 0.001). These results were consistent when performing subgroup analysis for unilateral and bilateral hernias.

**Conclusions:**

The benefits observed in terms of recurrence rates and post-surgical complications do not justify the additional costs incurred by the robotic approach to inguinal hernia within the national public healthcare system. Nevertheless, it represents a simpler way to initiate the robotic learning curve, justifying its use in a training context.

## Introduction

Inguinal hernia repair is one of the most common surgical procedures worldwide. Currently, the European Hernia Society (EHS) recommends minimally invasive surgery inguinal hernia repair over open surgery, especially in high-volume laparoscopic centers and selected cases. This is attributed long-term success, reduced chronic and immediate postoperative pain, shorter hospital stays, and quicker return to work and physical activity [1].

Since its first description in 2007, robotic inguinal hernia repair has experienced exponential growth, particularly in the USA, despite the associated high costs [2, 3]. This is because the robot offers several advantages over laparoscopy, including camera stability, improved tissue visualization, a wider range of motion, precision, and enhanced surgeon ergonomics [4, 5].

There are few studies comparing the robotic and laparoscopic approaches, most of which have small sample sizes in the robotic arm, limiting their generalizability. Nevertheless, it has been observed that both approaches yield similar results in terms of recurrence, chronic and postoperative pain, hospital stay, and return to normal activities [6–11]. The robotic approach does tend to have longer operating times compared to laparoscopy, although this difference often evens out as surgeons become more experienced [4, 9, 10].

In contrast, the cost of robotic inguinal hernia repair is significantly higher than laparoscopic repair, and the results are fairly consistent across different studies. The cost differential depends on the location (country and healthcare system) and the methodology used, including which expenses are analyzed, as some studies include the cost of robotic platforms while others do not [12–14].

The objective of this study is to conduct a comprehensive cost analysis in our setting (the Spanish National Public Healthcare System) comparing robotic and laparoscopic inguinal hernia repair and provide a cost-effectiveness analysis of both approaches.

## Methods

We conducted a retrospective single-center comparative study on inguinal hernia repair using a robotic or laparoscopic approach. The study was carried out at the Department of Surgery of a tertiary-level care center in Spain after approval of local ethics committee. Since our center started robotic surgery for hernia repair in 2021, all surgeries were performed by surgeons with a minimum of 4 years of experience in laparoscopic inguinal hernia repair. All surgeons included in this study have laparoscopic inguinal hernia experience of at least 5 years and are accredited to perform robotic surgery as a primary surgeon. Our robotic experience is limited because the robotic abdominal wall surgery repair program at our center began in October 2021.

Patients over 18 years of age with unilateral or bilateral inguinal hernias who underwent elective surgery using the robotic transabdominal preperitoneal approach (r-TAPP) from October 2021 to July 2023 were compared to patients who underwent elective laparoscopic transabdominal preperitoneal surgery (l-TAPP) during the same time period. Matching was done based on age, gender, and type of inguinal hernia. All patients signed the informed consent prior to surgery. Patients under the age of 18, those undergoing urgent surgery, open surgery, or a totally extraperitoneal (TEP) approach, and those with concurrent medical conditions were excluded. A standard 30-day follow-up was conducted at the clinic.

The total procedure cost was calculated using a micro-costing approach for direct costs of procedural and hospitalization. Indirect costs, related to the maintenance of the healthcare system, are difficult to calculate and are incurred independently of the chosen approach, so they are similar for both. In our case, we included the cost of the robotic and laparoscopic platforms used as indirect costs. Similarly, indirect cost associated whit post-surgical recovery (resumption of physical and occupational activities) are challenging to calculate.

**Fig. 1:**
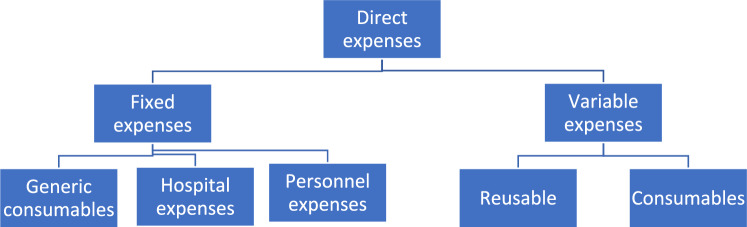
Cost categories included in the analysis

Direct costs were defined as the cost of resources directly used in the procedure and included two main categories: fixed and variable costs. Fixed costs encompass expenses related to hospitalization, which in our setting is a fixed amount of €613.96 per day of hospital stay. These costs cover nursing and other healthcare personnel, as well as generic consumables (medication, IV lines, and catheters), and hospitality costs (meals, cleaning, and accommodation). Variable costs include procedural costs, such as reusable instruments (forceps, light sources, etc.) and consumables used in the procedure (meshes, fixation, trocars, sterile bags, etc.). And the room occupancy cost has been estimated at 187€ per hour. The cost distribution can be observed in Fig. 1. The cost relation of consumables is shown in Table 1.

**Table 1:** Cost of materials consumables

	Cost
Robotic consumable	Trocars 34.49€ per unit (3 units) Obturator 250.59€ per use Bipolar dissector 329.11€ per use Bipolar cap 38.36€ per unit Scissor 614.68€ per use Robotic needle driver 251.68€ per use Arm protector 99.22€ per unit (3 units) Column protector 68.72€ per unit
Laparoscopic consumable	Atraumatic Grassper 38.65€ per unit Monopolar Dissector 37.47€ per unit Monopolar Scissor 20.5€ per unit Endoscopic needle driver reusable^*^ Trocars 5 mm 20.31€ per unit (2 units) Trocars 11 mm 30.80€ per unit Endoscopic sheath 5.15€ per unit
Other surgical consumables	Mesh (3D MAX) 143.00€ per unit Absorbable Tackers 220.00 € per unit Tissucol 330.00€ per unit Gauze 0.27€ per pack Bipolar 3.02€ per unit Stapler 3.01€ per unit Multifilament synthetic absorbable suture 2.14€ per unit Multifilament synthetic absorbable suture v-lock 30.05€ per unit Veress needle 10.60€ per unit Tube Endosuflator CO_2_ 53.02 € per unit
Inpatient stay	613.96€ stay per day
Operating room occupancy	185€ per hour

^a^These instruments is reusable, therefore, we do not considered the use-cost

Procedural-cost includes those associates with the intervention itself (robotic, laparoscopic, and general consumables) along with the operating room occupancy cost. Meanwhile, hospitalization-cost will encompass the cost of inpatient stay and the procedural-cost.

Other variables analyzed included demographic variables (age, gender, body mass index (BMI), American Society of Anesthesiologists (ASA) anesthesia risk classification), total hospital stay, surgical time, conversion rate, postoperative complications according to the Clavien–Dindo classification, and the 30-day readmission rate, recurrence at 1 month, 6 months and 1 year, pain measured using the visual analog pain scale (VAS) at 1 month, 6 months and 1 year, return to works (days) and return physical activity (days).

Patient characteristics were summarized using continuous and categorical variables. Continuous variables were presented as mean ± standard deviation (SD). Categorical variables were presented as frequency and percentage (%). Statistical analysis for categorical variables was performed using the Chi-square test, while the Student’s *T*-test was used for normally distributed continuous variables, and the Mann–Whitney *U* test or Kruskal–Wallis test for non-normally distributed samples. A *p*-value < 0.05 was considered statistically significant. Data analysis was performed using IBM® SPSS® Statistics 21.

## Results

**Fig. 2:**
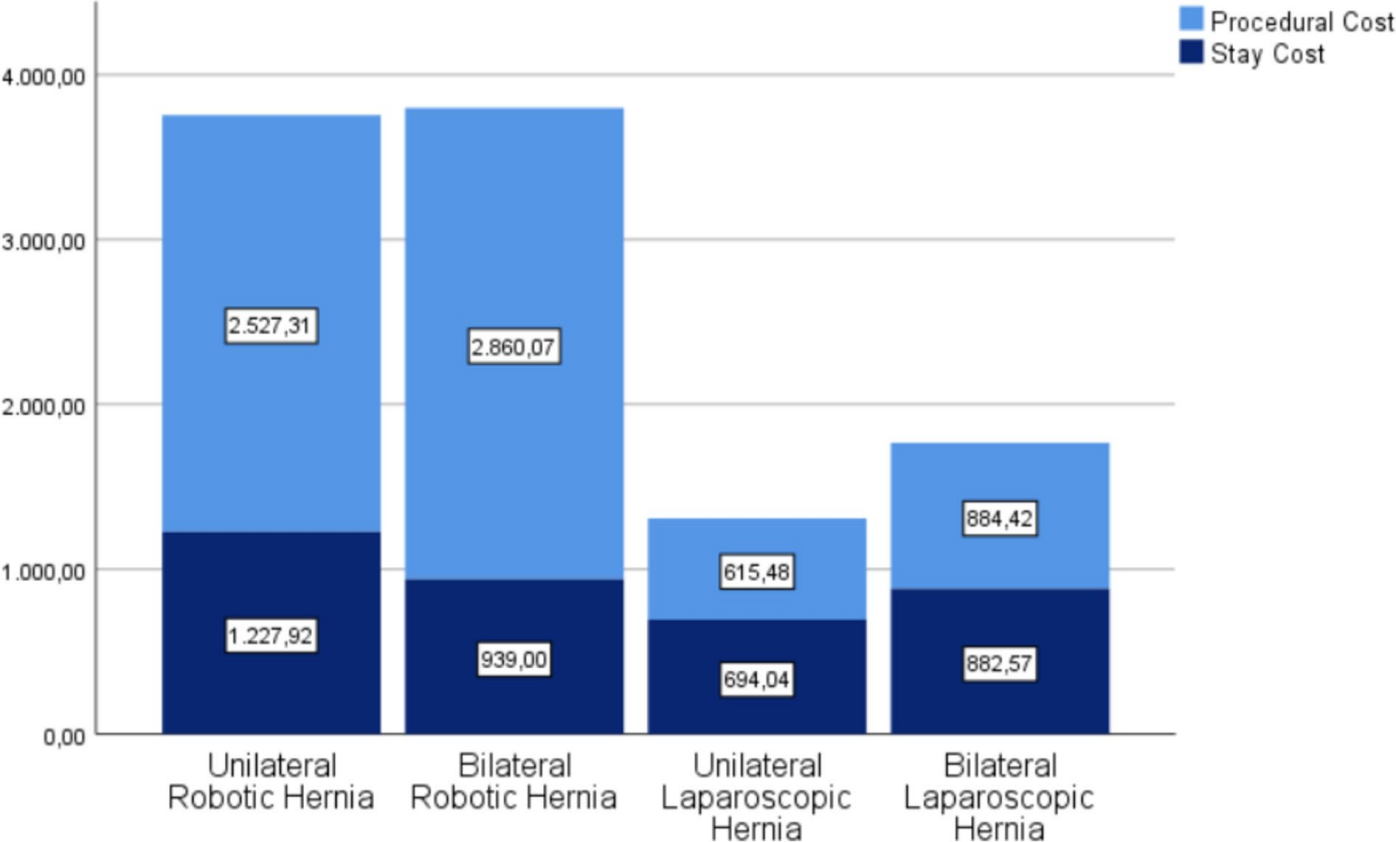
Cost analysis comparison between (robotic–laparoscopic) groups

The study included a total of 98 patients who underwent either robotic or laparoscopic TAPP inguinal hernia repair between October 2021 and July 2023. Out of these 98 patients, 20 (20.4%) underwent robotic TAPP, and 78 (79.6%) underwent laparoscopic TAPP. When comparing both approaches, no significant differences were found in the distribution by gender, age, BMI, ASA classification, recurrent hernias, postoperative complications (Clavien–Dindo classification), conversion rates, recurrence, unplanned readmission, or emergency department evaluation. The results are shown in Table 2 and Fig. 2.

**Table 2:** Summary of patient characteristics

	Robotic	Laparoscopic	
*Demographics*			
Gender	F = 1 (5%) M = 19 (95%)	F = 17 (21.8%) M = 19 (78.2%)	*p* = 0.84
Age (years)	66.10 ± 11.48	62.38 ± 13.88	*p* = 0.273
BMI (kg/m^2^)	26.70 ± 3.16	27.32 ± 4.09	*p* = 0.226
ASA	I = 1 (5%) II = 14 (70%) III = 5 (25%) IV = 0	I = 6 (7.8%) II = 56 (72.7%) III = 15 (19.5%) IV = 0	*p* = 0.81
*Operative variables*			
Hernia type	Unilateral = 3 (15%) Bilateral = 17 (85%)	Unilateral = 46 (59%) Bilateral = 32 (41%)	*p* < 0.01
Previous recurrence	Yes = 10 (50%) No = 10 (50%)	Yes = 33 (42.3%) No = 45 (57.7%)	*p* = 0.469
Number of previous recurrences	0.85 ± 1.39	0.94 ± 0.73	*p* = 0.741
Type of previous intervention	PHS = 0 Lichtenstein = 5 (50%) Herniorraphy = 3 (30%) Unknown = 2 (20%)	PHS = 9 (27.27%) Lichtenstein = 11 (33.33%) Herniorraphy = 4 (12.12%) Unknown = 9 (27.27%)	*p* = 0.212
Surgical time (min)	86 ± 33.07	40 ± 14.46	*p* < 0.001
Conversion	Yes = 0 No = 20 (100%)	Yes = 0 No = 78 (100%)	
Hospital stays (days)	1.6 ± 0.503	1.26 ± 0.49	*p* = 0.007
Complications (Clavien-Dindo)	0 = 20 (100%) I = 0 II = 0 III = 0 IV = 0 V = 0	0 = 76 (97.4%) I = 2 (2.6%) II = 0 III = 0 IV = 0 V = 0	*p* = 0.469
*Follow-up variables*			
Unplanned readmission	Yes = 0 No = 20 (100%)	Yes = 1 (1.3%) No = 77 (98.7%)	*p* = 0.611
Visit to emergency	Yes = 1 (5%) No = 19 (95%)	Yes = 2 (2.6%) No = 76 (97.4%)	*p* = 0.573
Reintervention	Yes = 0 No = 20 (100%)	Yes = 1 (1.3%)^a^ No = 77 (98.7%)	*p* = 0.611
Surgical site complications	No = 14 (70%) SSO (hematoma/seroma) = 6 (30%) SSI (infection) = 0	No = 55 (70.5%) SSO (hematoma/seroma) = 22 (28.2%) SSI (infection) = 1 (1.3%)	*p* = 0.328
Recurrence 1 month	Yes = 0 No = 20 (100%)	Yes = 1 (1.3%)^a^ No = 77 (98.7%)	*p* = 0.611
Recurrence at 6 months *N* = 97	Yes = 0 No = 20 (100%)	Yes = 1 (1.3%) No = 76 (98.7%)	*p* = 0.608
Recurrence at 1 years *N* = 94	Yes = 0 No = 20 (100%)	Yes = 1 (1.4%) No = 73 (98.6%)	*p* = 0.601
Postoperative pain (VAS)	2.00 ± 1.84	1.50 ± 1.87	*p* = 0.404
Pain at 1 month	No = 16 (80%) Non-neuropathic = 4 (20%) Neuropathic = 0	No = 63 (80.8%) Non-neuropathic = 10 (12.8%) Neuropathic = 5 (6.4%)	*p* = 0.395
Pain at 6 months *N* = 89	No = 14 (87.5%) Non-neuropathic = 2 (12.5%) Neuropathic = 0	No = 63 (86.3%) Non-neuropathic = 4 (5.5%) Neuropathic = 6 (8.2%)	*p* = 0.320
Pain at 1 year *N* = 82	No = 14 (87.5%) Non-neuropathic = 2 (12.5%) Neuropathic = 0	No = 55 (83.3%) Non-neuropathic = 4 (6.1%) Neuropathic = 7 (8.5%)	*p* = 0.293
Return to work (days)	54.29 ± 36.9	128.10 ± 78.73	*p* = 0.481
Return to physical activity (days)	16.20 ± 10.54	13.33 ± 13.53	*p* = 0.453
Cost			
Procedure cost (€)	2810.15 ± 218.10	725.81 ± 158.91	*p* < 0.001
Stay cost (€)	982.33 ± 308.59	771.38 ± 303.94	*p* < 0.007
Hospitalization cost (€)	3792.49 ± 326.06	1497.20 ± 375.68	*p* < 0.001

^a^Intervention for interstitial hernia due to flap opening

We can observe significant differences in surgical time (86 ± 33.07 min vs. 40 ± 14.46 min (95% CI: 35.28–54.79), *p* < 0.001), and in hospital stay (1.6 ± 0.503 days vs. 1.26 ± 0.49 days, (95% CI: 0.097–0.591), *p* < 0.007), which are longer in patients treated through the robotic approach.

Regarding the costs, both the price of robotic procedural (2810.15€ ± 218.10€ vs. 725.81€ ± 158.91€ (95% CI: 1998.64–2170.04), *p* < 0.001) and hospitalization robotic costs (3792.49€ ± 326.06€ vs. 1497.20€ ± 375.68€ (95% CI: 2113.01–2477.58), *p* < 0.001) are significantly higher than in the laparoscopic approach.

When we perform a subgroup analysis and compare the results of unilateral robotic hernias versus laparoscopic ones, we obtain similar results. No significant differences are observed between the analyzed groups (Age, ASA, Gender, BMI). There are no differences in postoperative complications, no conversions or readmissions have occurred, and the recurrence rate and surgical site complications are similar in both subgroups. The data are detailed in Table 3.

**Table 3:** Comparison between unilateral robotic and laparoscopic approaches

Unilateral	Robotic	Laparoscopic	
*Demographics*			
Gender	F = 1 (33.3%) M = 2 (66.6%)	F = 13 (28.3%) M = 33 (71.7%)	*p* = 0.851
Age (years)	68.67 ± 16.42	62.91 ± 15.68	*p* = 0.542
BMI (kg/m^2^)	24.45 ± 3.61	26.75 ± 4.18	*p* = 0.359
ASA	I = 0 II = 3 (100%) III = 0 IV = 0	I = 4 (8.9%) II = 34 (75.6%) III = 7 (15.6%) IV = 0	*p* = 0.621
*Operative variables*			
Previous recurrence	Yes = 1 (33.3%) No = 2 (66.6%)	Yes = 18 (39.13%) No = 28 (60.87%)	*p* = 0.9
Number of previous recurrence	0.67 ± 1.15	0.73 ± 0.691	*p* = 0.881
Type of previous intervention	PHS = 0 Lichtenstein = 0 Herniorraphy = 0 Unknown = 1 (100%)	PHS = 6 (33.33%) Lichtenstein = 7 (38.88%) Herniorraphy = 3 (16.66%) Unknown = 2 (11.11%)	*p* = 0.295
Surgical time (min)	50 ± 25.98	33.37 ± 9.78	*p* = 0.014
Conversion	Yes = 0 No = 3 (100%)	Yes = 0 No = 46 (100%)	
Hospital stays (days)	2 ± 0.001	1.11 ± 0.379	*p* < 0.001
Complications (Clavien-Dindo)	0 = 3 (100%) I = 0 II = 0 III = 0 IV = 0 V = 0	0 = 45 (97.8%) I = 1 (2.2%) II = 0 III = 0 IV = 0 V = 0	*p* = 0.796
*Follow-up variables*			
Unplanned readmission	Yes = 0 No = 3 (100%)	Yes = 0 No = 46 (100%)	
Visit to emergency	Yes = 0 (0%) No = 3 (100%)	Yes = 2 (4.3%) No = 44 (95.7%)	*p* = 0.712
Reintervention	Yes = 0 No = 3 (100%)	Yes = 0 No = 46 (100%)	
Surgical site complications	No = 3 (100%) SSO (hematoma/seroma) = 0 SSI (infection) = 0	No = 33 (71.7%) SSO (hematoma/seroma) = 12 (26.1%) SSI (infection) = 1 (2.2%)	*p* = 0.842
Recurrence 1 month	Yes = 0 No = 3 (100%)	Yes = 1 (2.2%)^a^ No = 45 (97.8%)	*p* = 0.796
Recurrence at 6 months	Yes = 0 No = 3 (100%)	Yes = 0 No = 45 (100%)	
Recurrence at 1 years	Yes = 0 No = 20 (100%)	Yes = 0 No = 45 (100%)	
Postoperative pain (VAS)	0.67 ± 0.58	0.83 ± 0.93	*p* = 0.798
Pain at 1 month	No = 2 (66.6%) Non-neuropathic = 1 (33.3%) Neuropathic = 0	No = 40 (87%) Non-neuropathic = 3 (6.5%) Neuropathic = 3 (6.5%)	*p* = 0.245
Pain at 6 months *N* = 45	No = 3 (100%) Non-neuropathic = 0 Neuropathic = 0	No = 38 (90.5%) Non-neuropathic = 1 (2.4%) Neuropathic = 3 (7.1%)	*p* = 0.855
Pain at 1 year *N* = 40	No = 3 (100%) Non-neuropathic = 0 Neuropathic = 0	No = 33 (89.2%) Non-neuropathic = 1 (2.7%) Neuropathic = 3 (8.1%)	*p* = 0.835
Return to work (days)	30.5 ± 0.707	105.78 ± 166.58	*p* = 0.212
Return to physical activity (days)	18.50 ± 11.50	14.09 ± 15.62	*p* = 0.767
*Cost*			
Procedure cost (€)	2527.31 ± 45.81	615.47 ± 73.33	*p* < 0.001
Stay cost (€)	1227.92 ± 0.001	694.04 ± 245.88	*p* < 0.001
Total cost (€)	3755.22 ± 45.81	1309.52 ± 249.76	*p* < 0.001

^a^Intervention for interstitial hernia due to flap opening

In this subgroup analysis (unilateral inguinal hernia), we can also observe that the surgical time (50 ± 25.98 min vs. 33.37 ± 9.78 min (95% CI: 3.48–29.77), *p* < 0.014) and the operative stay (2 ± 0.001 days vs. 1.11 ± 0.379 days (95% CI: 0.447–1.336), *p* < 0.001) are significantly longer in the robotic subgroup.

Regarding the costs, we can observe that the expense of the robotic procedure is significantly higher than the laparoscopic procedure. This applies to both the intervention costs (2527.31€ ± 45.81€ vs. 615.47€ ± 73.33€ (95% CI: 1825.07–1998–59), *p* < 0.001) and the hospitalization costs (3755.22 ± 45.81€ vs. 1309.52 ± 249.76€ (95% CI: 2152.52–2738.89), *p* < 0.001).

Finally, when performing a subgroup analysis of bilateral hernias, we observe similar results. No differences are noted in demographic variables or postoperative complications, conversion rate, recurrence, or surgical site complications. The results are shown in Table 4.

**Table 4:** Comparison between robotic and laparoscopic bilateral hernias

Bilateral	Robotic	Laparoscopic	
*Demographics*			
Gender	F = 0 M = 17 (100%)	F = 4 (12.5%) M = 28 (87.5%)	*p* = 0.853
Age (years)	65.65 ± 11.01	61.63 ± 11.01	*p* = 0.230
BMI (kg/m^2^)	27.10 ± 3.03	28.16 ± 3.87	*p* = 0.232
ASA	I = 1 (5.9%) II = 11 (64.7%) III = 5 (29.4%) IV = 0	I = 2 (6.3%) II = 22 (68.8%) III = 8 (25%) IV = 0	*p* = 0.621
*Operative variables*			
Previous recurrence	Yes = 9 (52.9%) No = 8 (47.1%)	Yes = 15 (46.9%) No = 17 (53.1%)	*p* = 0.686
Number of previous recurrence	0.88 ± 1.45	1.29 ± 0.686	*p* = 0.299
Type of previous intervention	PHS = 0 Lichtenstein = 5 (55.55%) Herniorraphy = 3 (33,33%) Unknown = 1 (11.11%)	PHS = 6 (40%) Lichtenstein = 7 (46.66%) Herniorraphy = 3 (20%) Unknown = 2 (13.33%)	*p* = 0.091
Surgical time (min)	92.35 ± 30.47	51.88 ± 13.12	*p* < 0.001
Conversion	Yes = 0 No = 17 (100%)	Yes = 0 No = 32 (100%)	
Hospital stays (days)	1.53 ± 0.514	1.16 ± 1.05	*p* = 0.337
Complications (Clavien-Dindo)	0 = 17 (100%) I = 0 II = 0 III = 0 IV = 0 V = 0	0 = 31 (96.9%) I = 1 (3.1%) II = 0 III = 0 IV = 0 V = 0	*p* = 0.796
*Follow-up variables*			
Unplanned readmission	Yes = 0 No = 17 (100%)	Yes = 0 No = 32 (100%)	
Visit to emergency	Yes = 1 (5.9%) No = 16 (94.1%)	Yes = 0 No = 32 (100%)	*p* = 0.712
Reintervention	Yes = 0 No = 17 (100%)	Yes = 0 No = 32 (100%)	
Surgical site complications	No = 11 (64.7%) SSO (hematoma/seroma) = 6 (35.3%) SSI (infection) = 0	No = 22 (69.8%) SSO (hematoma/seroma) = 10 (32.2%) SSI (infection) = 0	*p* = 0.12
Recurrence 1 month	Yes = 0 No = 17 (100%)	Yes = 0 No = 32 (100%)	
Recurrence at 6 months *N* = 48	Yes = 0 No = 17 (100%)	Yes = 1 (3.2%) No = 31 (96.8%)	*p* = 0.454
Recurrence at 1 year *N* = 46	Yes = 0 No = 17 (100%)	Yes = 1 (3.4%) No = 28 (96.6%)	*p* = 0.439
Postoperative pain (VAS)	2.36 ± 1.91	1.79 ± 2.12	*p* = 0.487
Pain at 1 month	No = 14 (86.4%) Non-neuropathic = 3 (17.6%) Neuropathic = 0	No = 23 (71.9%) Non-neuropathic = 7 (21.9%) Neuropathic = 2 (6.3%)	*p* = 0.517
Pain at 6 months *N* = 44	No = 11 (84.6%) Non-neuropathic = 2 (15.4%) Neuropathic = 0	No = 25 (80.6%) Non-neuropathic = 3 (9.7%) Neuropathic = 3 (9.7%)	*p* = 0.463
Pain at 1 year *N* = 42	No = 11 (84.6%) Non-neuropathic = 2 (15.4%) Neuropathic = 0	No = 22 (75.9%) Non-neuropathic = 3 (10.3%) Neuropathic = 4 (13.8%)	*p* = 0.355
Return to work (days)	58.33 ± 38.68	66,67 ± 121.13	*p* = 0.653
Return to physical activity (days)	15.85 ± 10.32	11.89 ± 8.68	*p* = 0.257
Cost			
Procedure cost (€)	2860.06 ± 196.41	884.42 ± 104.35	*p* < 0.001
Stay cost (€)	938.99 ± 315.88	882.56 ± 346.51	*p* = 0.579
Total cost (€)	3799.06 ± 354.52	1766.98 ± 364.16	*p* < 0.001

Unlike unilateral hernias, when we analyze the operative stay between robotic and laparoscopic approaches in bilateral hernia, we do not observe statistical differences between them (1.53 ± 0.514 days vs. 1.16 ± 1.05 days (95% CI:  − 0.173–0.919), *p* = 0.337). However, we do see differences in surgical time, which remains longer in the robotic arm compared to the laparoscopic arm (92.35 ± 30.47 min vs. 51.88 ± 13.12 min (95% CI: 27.56–52.99), *p* < 0.001).

Regarding the costs, we can observe that the expense of the robotic procedure is significantly higher than the laparoscopic procedure. This applies to both the intervention costs (2860.06 ± 196.41€ vs. 884.42 ± 104.35€ (95% CI: 1889.59–2061.70), *p* < 0.001) and the hospitalization costs (3799.06 ± 354.52€ vs. 1766.98 ± 364.16€ (95% CI: 1814.17–2249.97), *p* < 0.001).

## Discussion and conclusions

The cost of surgical technologies is an increasingly analyzed variable, as healthcare systems have limited resources and must use their budget responsibly [15].

The cost-effectiveness of robotic surgery has been a subject of debate since its inception, with proponents highlighting its ergonomic and visual benefits and its ability to access limited areas, while critics emphasize the high associated cost [2–5].

In our study, we observed that robotic inguinal hernia repair had an approximate additional cost of €2084.34 compared to laparoscopic repair. This cost increase is similar to what has been observed in other studies in the United States, where differences range from $926 to $3999 [8, 12, 13, 16], although it is higher than the cost analysis in European Union studies (Belgium) with an average cost increase of €649 [14]. These differences may be attributed to the heterogeneity of healthcare systems, especially within Europe. In Spain, the healthcare system is universal and free, and although open hernia repair is commonly performed on an outpatient basis, laparoscopic hernia repair is not yet widespread, and robotic programs are still in the early stages of implementation in our country.

Considering the cost-effectiveness of both surgical approaches, the indirect costs due to lost workdays were not analyzed in our study. Nevertheless, we believe that while postoperative recovery is similar, with similar pain levels, and data related to resuming daily activities are also comparable, the indirect costs due to lost workdays do not appear to be determinative in this analysis.

Postoperative outcomes (recurrence, conversion, postoperative complications) are similar in both groups, with excellent results. This is consistent with findings from various studies, which show that the robotic transabdominal preperitoneal approach (r-TAPP) is safe and reproducible, with outcomes similar to laparoscopic TAPP (l-TAPP) [10, 11, 13, 17].

However, the operative time and hospital stay are longer for patients undergoing robotic procedures. This may be due to the initial surgeries performed as part of the learning curve for the robotic platform [4, 18, 19]. When we conducted a subgroup analysis based on unilateral inguinal hernias, we observed that in the case of bilateral hernias, these differences tended to decrease, and statistical significance was even lost for the length of hospital stay. These slight differences between unilateral and bilateral hernias justify the prioritization of bilateral hernias over unilateral ones when selecting patients for robotic surgery.

One limitation of our study is its retrospective nature and the lack of patient randomization, which may introduce selection bias. We attempted to mitigate this potential bias by matching patients for complexity and type of hernia, resulting in no significant differences between groups. Nevertheless, in our robotic group, we found a 50% recurrence rate, with most of them being bilateral hernias. Although these results were not statistically significant, they indicate a trend toward performing more complex hernia cases using robotics due to the advantages it offers in dissection and visualization of a previously operated area. This suggests that once the initial learning curve is overcome, these complex cases are the ones that benefit the most from robotic surgery.

Another drawback of our study is that it does not take into account the initial investment required to acquire robotic and laparoscopic devices, which can sometimes be challenging to amortize. Therefore, this cost, when added to the cost of robotic consumables, makes performing inguinal hernia repair using a robotic approach neither cost-efficient nor sustainable for the healthcare system. On the other hand, having both platforms in our hospital for performing other procedures does not imply an extra cost for the system.

Furthermore, the current cost of robotic platforms and the consumables used for surgical procedures have decreased in recent years, with a presumed trend towards further reductions due to the entry of new companies into this field and the expansion of robotic surgery in our environment. We hope that in the medium and long term, there will be a reduction in prices that could facilitate the implementation of robotic inguinal hernia repair.

A reduction in complications or recurrences could be a reason to justify this extra cost. Despite a trend toward better outcomes in terms of complications in the robotic group, we have not seen a statistically significant difference in the various studies published in the literature [10, 11, 13, 17], including ours, to justify the excess cost. These results may be related to the small sample size in the robotic surgery group, which could overestimate the cost of the procedure and undervalue the better clinical outcomes.

Likewise, there are intangible parameters that cannot be economically assessed and are, therefore, not included in the cost-effectiveness analysis. These parameters include ergonomic and visual improvements for the surgeon, increased safety in performing various surgical maneuvers, technological advancement and innovation provided by the robotic platform, as well as the necessary learning curve to perform more complex procedures, which can justify the use of the robotic platform. In the realm of robotic surgery, inguinal hernia repair is considered by some experts as the “new laparoscopic cholecystectomy” in terms of training. It is considered the ideal starting point for robotic surgical training because it requires all the dissection, manipulation, visualization, and suturing skills necessary to initiate training, similar to what laparoscopic cholecystectomy represented in the early days of laparoscopic surgery.

In conclusion, the cost of robotic surgical instruments seems to be too high, and the benefits are too limited to allow for widespread use of robotic inguinal hernia repair in a European Union context, and more specifically, in Spain. However, we believe that robotic surgery may have benefits for complex hernias (bilateral and recurrent) due to the advantages offered by the robotic platform. In addition, the training aspect of inguinal hernia repair on the robotic learning curve is a valuable starting point for more complex procedures.
